# Gα12 regulates osteoclastogenesis by modulating NFATc1 expression

**DOI:** 10.1111/jcmm.13370

**Published:** 2017-10-27

**Authors:** Min‐Kyoung Song, Cheolkyu Park, Yong Deok Lee, Haemin Kim, Min Kyung Kim, Jun‐Oh Kwon, Ja Hyun Koo, Min Sung Joo, Sang Geon Kim, Hong‐Hee Kim

**Affiliations:** ^1^ Department of Cell and Developmental Biology BK21 Program and Dental Research Institute Seoul National University Seoul Korea; ^2^ College of Pharmacy and Research Institute of Pharmaceutical Sciences Seoul National University Seoul Korea; ^3^ Present address: Department of Surgery and Cancer Research Institute Seoul National University College of Medicine Seoul Korea

**Keywords:** G alpha 12, nuclear factor of activated T‐cell c1, osteoclast, receptor activator of nuclear factor κB ligand, RhoA

## Abstract

The G12 family of G protein alpha subunits has been shown to participate in the regulation of various physiological processes. However, the role of Gα12 in bone physiology has not been well described. Here, by micro‐CT analysis, we discovered that Gα12‐knockout mice have an osteopetrotic phenotype. Histological examination showed lower osteoclast number in femoral tissue of Gα12‐knockout mice compared to wild‐type mice. Additionally, *in vitro* osteoclastic differentiation of precursor cells with receptor activator of nuclear factor‐κB ligand (RANKL) showed that Gα12 deficiency decreased the number of osteoclast generated and the bone resorption activity. The induction of nuclear factor of activated T‐cell c1 (NFATc1), the key transcription factor of osteoclastogenesis, and the activation of RhoA by RANKL was also significantly suppressed by Gα12 deficiency. We further found that the RANKL induction of NFATc1 was not dependent on RhoA signalling, while osteoclast precursor migration and bone resorption required RhoA in the Gα12‐mediated regulation of osteoclasts. Therefore, Gα12 plays a role in differentiation through NFATc1 and in cell migration and resorption activity through RhoA during osteoclastogenesis.

## Introduction

Bone homeostasis is exquisitely maintained by a harmony of bone‐forming osteoblasts and bone‐resorbing osteoclasts. Abnormal increases in the number or excessive stimulation of the activity of osteoclasts are often related to erosive bone diseases, such as osteoporosis, or inflammatory bone lysis. Osteoclasts are multinuclear giant cells that are differentiated from the monocyte/macrophage lineage of hematopoietic cells. For osteoclastogenesis, two major cytokines are required: macrophage colony‐stimulating factor (M‐CSF) and receptor activator of nuclear factor κB ligand (RANKL). M‐CSF supports the survival and proliferation of precursor cells, and RANKL induces their differentiation into osteoclasts 1. The binding of RANKL to the RANK receptor recruits tumour necrosis factor receptor‐associated factor 6 (TRAF6) and brings together the signalling molecules required for the activation of mitogen‐activated protein kinase (MAPK) or NF‐κB 2, 3. RANKL also induces and activates the c‐Fos transcription factor and activates calcium signalling in cooperation with additional cell surface immune receptors. Eventually, these signalling pathways activate nuclear factor of activated T‐cell c1 (NFATc1), the transcription factor regarded as a master regulator of osteoclastogenesis, and drives the expression of the osteoclast marker genes such as tartrate‐resistant acid phosphatase (TRAP), cathepsin K and dendritic cell‐specific transmembrane protein (DC‐STAMP) 4. When osteoclasts become mature and functionally activated, they resorb the bone by secreting bone‐degrading enzymes in resorption lacunae.

Heterotrimeric G proteins are classified into four subfamilies based on their alpha subunits: Gs, Gi, Gq and G12 5, 6. The most recently identified G12 subfamily consists of Gα12 and Gα13, which are ubiquitously expressed in various cells and tissues 7. The first identified function of Gα12 was oncogenic transformation 8; since then, Gα12 has been reported to regulate multiple cellular responses including growth, polarity, migration and apoptosis in various physiological processes, such as embryonic development, angiogenesis, platelet activation, and immune and neuronal actions 9, 10, 11. The Gα12‐mediated signalling response has mainly been described for small GTPase Rho activation through RhoGEF, which has been linked to the regulation of actin stress fibres and the induction of transcriptional activity 12, 13, 14. Other than RhoGEF, more than 20 proteins have been revealed as effector molecules for Gα12, such as cadherin, Hsp90 and BTK 15, 16, 17. This implies that Gα12 could function through diverse mechanisms in different tissues.

Gα12 shows a 67% amino acid identity with Gα13, and they share many of the same binding G protein‐coupled receptors (GPCRs) and downstream effector molecules. However, the presence of significant differences between Gα12 and Gα13 has been demonstrated by knockout mice studies. Gα12‐deficient mice are viable, fertile and show no noticeable gross abnormalities 18, 19. In striking contrast, Gα13‐deficient mice are embryonic lethal due to angiogenic defects. The skeletal phenotypes of Gα12‐knockout mice have not been investigated in detail.

In this study, we found increased trabecular bone volume in Gα12‐knockout mice, and this result prompted us to investigate the function of Gα12 in osteoclastogenesis. Here, we report for the first time the bone phenotype of Gα12‐knockout mice and suggest that Gα12 has a crucial role in RANKL‐induced osteoclastogenesis.

## Materials and Methods

### Reagents

Recombinant human M‐CSF and human soluble RANKL were purchased from PeproTech (Rocky Hill, NJ, USA). Anti‐NFATc1 (7A6) and anti‐c‐Fos (H125) antibodies were from Santa Cruz Biotechnology (Santa Cruz, CA, USA), and monoclonal antibodies against β‐actin (AC‐74) and secondary antibodies were purchased from Sigma‐Aldrich (St. Louis, MO, USA). Antibodies against ERK, JNK, p38, IκB, and Akt were from Cell Signaling Technology (Cambridge, MA, USA) as were phospho‐specific antibodies for ERK (Thr202/Tyr204), JNK (Thr182/Tyr185), p38 (Thr180/Tyr182), IκB (Ser32) and Akt (Ser473). All other reagents were obtained from Sigma‐Aldrich.

### Bioinformatic analysis

Microarray data were downloaded from Gene Expression Omnibus (GSE57468). Differentially expressed genes (DEGs) upon osteoclast differentiation were identified independently using Student's *t*‐test: DEGs were selected as the genes having *P*‐values < 0.005 at every time‐points compared with control group. Statistically enriched signalling pathways of clustered DEGs were ranked and categorized according to Reactome pathways using DAVID 6.7 software (National Institutes of Health, Bethesda, MD). Hierarchical clustering with Pearson's correlation of the genes encoding Gα proteins was performed using R software with pvclust package (Bioconductor, http://www.r-project.org/). H3K27ac ChIP‐seq data were collected from ENCODE. ChIP‐seq data were analysed using Integrative Genomics Viewer (IGV) software (Broad Institute, Cambridge, MA).

### Mice and cell culture

Gα12^−/−^ mice have been described previously 18. Whole bone marrow cells were flushed from the tibias and femurs of 5‐week‐old ICR mice or 7‐week‐old Gα12^−/−^ mice. After the elimination of erythrocytes with hypotonic buffer, cells were incubated overnight in alpha‐modified Eagle's medium (α‐MEM) with 10% foetal bovine serum in culture dishes. Adherent cells were discarded, and floating cells were further cultured with 30 ng/ml M‐CSF on Petri dishes. After three days, adherent cells were scraped and regarded as bone marrow‐derived macrophages (BMMs). For the differentiation of osteoclasts, BMMs were cultured with 30 ng/ml M‐CSF and 120 ng/ml RANKL.

### siRNA transfection

BMMs were transfected with 40 nM control or Gα12‐specific siRNAs (Santa Cruz) with HiPerFect transfection reagent (Qiagen, Hilden, Germany). After overnight incubation, the culture medium was replaced with a fresh medium.

### Plasmid transfection and retroviral gene transfer

BMMs were transfected with pcDNA3‐EGFP or pcDNA3‐RhoA‐CA (constitutively active form) using PolyFect (Qiagen). Retroviral packaging was performed by transfecting Plat E cells with pMX‐puro or pMX‐puro‐hNFATc1CA using PolyFect. At 24 hrs after transfection, culture medium containing viral particles was collected and filtered through a 0.45‐μm syringe filter. For retroviral infection, BMMs were incubated in the virus‐containing medium with 4 μg/ml polybrene and 30 ng/ml M‐CSF for 24 hrs.

### μCT analysis

Femurs from 9‐week‐old WT and Gα12^−/−^ male mice were analysed with a SkyScan 1172 (SkyScan, Aartselaar, Belgium; 70 Kv, 141 μA, 6.92 pixel size). Trabecular bone was measured in the 1‐mm‐thick region starting 1 mm below the growth plate at thresholds of 89 minimum and 255 maximum. Bone parameters were calculated by a CT‐analyzer program (version 1.7; SkyScan), and three‐dimensional images were obtained by CT‐volume software (version 1.11; SkyScan).

### TRAP staining

Cells were fixed with 3.7% formaldehyde and permeabilized in 0.1% Triton X‐100 for 1 min. TRAP staining was performed for 5–15 min. using a commercial kit (Sigma‐Aldrich, Cat. No. 387A‐1KT) according to the manufacturer's instructions.

### Dentin resorption assay

BMMs were cultured on dentin slices for nine days in the presence of 120 ng/ml RANKL and 30 ng/ml M‐CSF. After the cells were removed with distilled water, dentin slices were observed under an LSM 5 Pascal confocal microscope (Carl Zeiss MicroImaging GmbH, Goettingen, Germany). Bone resorption area and depth were measured with image analysis software (LSM 5 Image Browser; Zeiss).

### Reverse transcription‐polymerase chain reaction (RT‐PCR)

Total RNA was isolated from cells using TRIzol reagent (Invitrogen, Carlsbad, CA, USA), and 3 μg RNA was used to synthesize cDNA using Superscript II reverse transcriptase (Invitrogen). Real‐time PCR was performed using a KAPA SYBR FAST qPCR Kit (Kapa Biosystems, Wilmington, MA, USA) and ABI7300 real‐time system (Applied Biosystems, Walmington, MA, USA). Thermocycling conditions were as follows: an initial activation step at 95°C for 3 min., followed by 40 cycles of denaturation at 95°C for 3 sec. and amplification at 60°C for 33 sec. The primer sequences used are listed in Table 1. All reactions were run in triplicate, and gene expression levels were determined as fold changes using the cycle threshold comparison method. The housekeeping gene HPRT was used for normalization.

**Table 1:** The primers used in PCR analysis

Primer name	Primer sequence 5′‐3′
Gα12	TATGACCAGGTCCTCATGGA CAGGAGGTCCATCTTGTTGA
18srRNA	GTAACCCGTTGAACCCCATT CCATCCAATCGGTAGTAGCG
Nfatc1	CCAGTATACCAGCTCTGCCA GTGGGAAGTCAGAAGTGGGT
Acp5	CGACCATTGTTAGCCACATACG TCGTCCTGAAGATACTGCAGGTT
Ctsk	ATATGTGGGCCACCATGAAAGTT TCGTTCCCCACAGGAATCTCT
Dcstamp	GGGTGCTGTTTGCCGCTG CGACTCCTTGGGTTCCTTGCT
Atp6v0d2	GGGAGACCCTCTTCCCCACC CCACCGACAGCGTCAAACAAA
HPRT	CCTAAGATGAGCGCAAGTTGAA CCACAGGGACTAGAACACCTGCTAA

### Histological analysis

Histological measurements were performed as previously described 20. Briefly, femurs were fixed in 4% paraformaldehyde and decalcified with 12% ethylenediaminetetraacetic acid (EDTA) for four weeks. Decalcified femurs were dehydrated and embedded in paraffin. Then, 5‐μm‐thick sections were stained for TRAP and with haematoxylin and were analysed using Osteomeasure software (Osteometrics, Decatur, CA, USA).

### Actin ring analysis

BMMs were cultured on 15‐mm glass coverslips and then fixed with 3.7% formaldehyde. Cells were permeabilized with 0.1% Triton X‐100, and non‐specific binding sites were blocked with 1% bovine serum albumin in phosphate‐buffered saline (PBS) for 90 min. After the cells were washed with PBS, they were stained with actin using rhodamine‐phalloidin for 2 hrs and mounted in the presence of 4′,6‐diamidino‐2‐phenylindole (DAPI). Images were obtained using a Zeiss LSM700 confocal microscope (Carl Zeiss).

### Transwell migration assay

BMMs were transfected with pcDNA3‐EGFP or RhoCA (the constitutively active form of RhoA) constructs with a polyfect transfection reagent (Qiagen). Transfected BMMs (1 × 10^5^) in serum‐free α‐MEM were transferred to the upper chamber of transwell plates with 8‐μm pores (Corning, Corning, NY, USA). The lower chambers were filled with serum‐free α‐MEM containing 240 ng/ml RANKL as an attractant. After 16 hrs of incubation in a CO_2_ incubator at 37°C, cells were fixed and stained with crystal violet.

### RhoA activity assay

Rho activity was measured using a Rho Activation Assay Kit (Merck Millipore, Darmstadt, Germany) according to the manufacturer's protocol. Briefly, after 3 hrs of serum starvation, BMMs were stimulated with 360 ng/ml RANKL and harvested at 0 and 15 min. Cells were lysed with lysis buffer and spun down, and then the supernatant was incubated with GTP‐Rho‐specific binding beads (containing Rhotekin Rho‐binding domain) at 4°C for 45 min. After the beads were washed three times, the samples were boiled with sample buffer. The supernatants of the boiled samples were loaded on 12°C sodium dodecyl sulphate (SDS)‐polyacrylamide gels, and immunoblotting was performed using anti‐Rho antibodies.

### BrdU assay

BrdU incorporation into cells was measured using a BrdU Cell Proliferation Assay Kit (Calbiochem, La Jolla, CA, USA) following the manufacturer's protocol.

### Western blotting

Cells were lysed with RIPA buffer [120 mM Tris‐HCl, pH 7.5, 150 mM NaCl, 1 mM Na_2_EDTA, 1 mM EGTA, 0.5% NP‐40, 2.5 mM sodium pyrophosphate, 1 mM β‐glycerophosphate, 1 mM Na_3_VO_4_, 1 mM NaF and a protease inhibitor cocktail (Roche, Mannheim, Germany)]. Protein concentrations of the cell lysates were measured using a DC Protein Assay Kit (Bio‐Rad, Hercules, CA, USA), and the same amounts of protein were loaded onto 10% SDS‐polyacrylamide gels for electrophoresis. After protein transfer onto nitrocellulose membranes (Amersham Pharmacia, Uppsala, Sweden), non‐specific binding sites on the membranes were blocked with 5% skim milk for 1 hr, and the membranes were incubated with primary antibodies overnight at 4°C. The membranes were then washed several times with Tris‐buffered saline with Tween‐20 and incubated with secondary antibodies conjugated with horseradish peroxidase in 2% skim milk for 1 hr. Immunoreactive bands were detected with electrochemiluminescent reagents in the dark.

### Statistical analysis

Statistical differences between the two groups were determined using Student's *t*‐test. P‐values <0.05 were regarded as significant.

## Results

### Decrease in Gα12 transcript level during osteoclast differentiation

**Figure 1:**
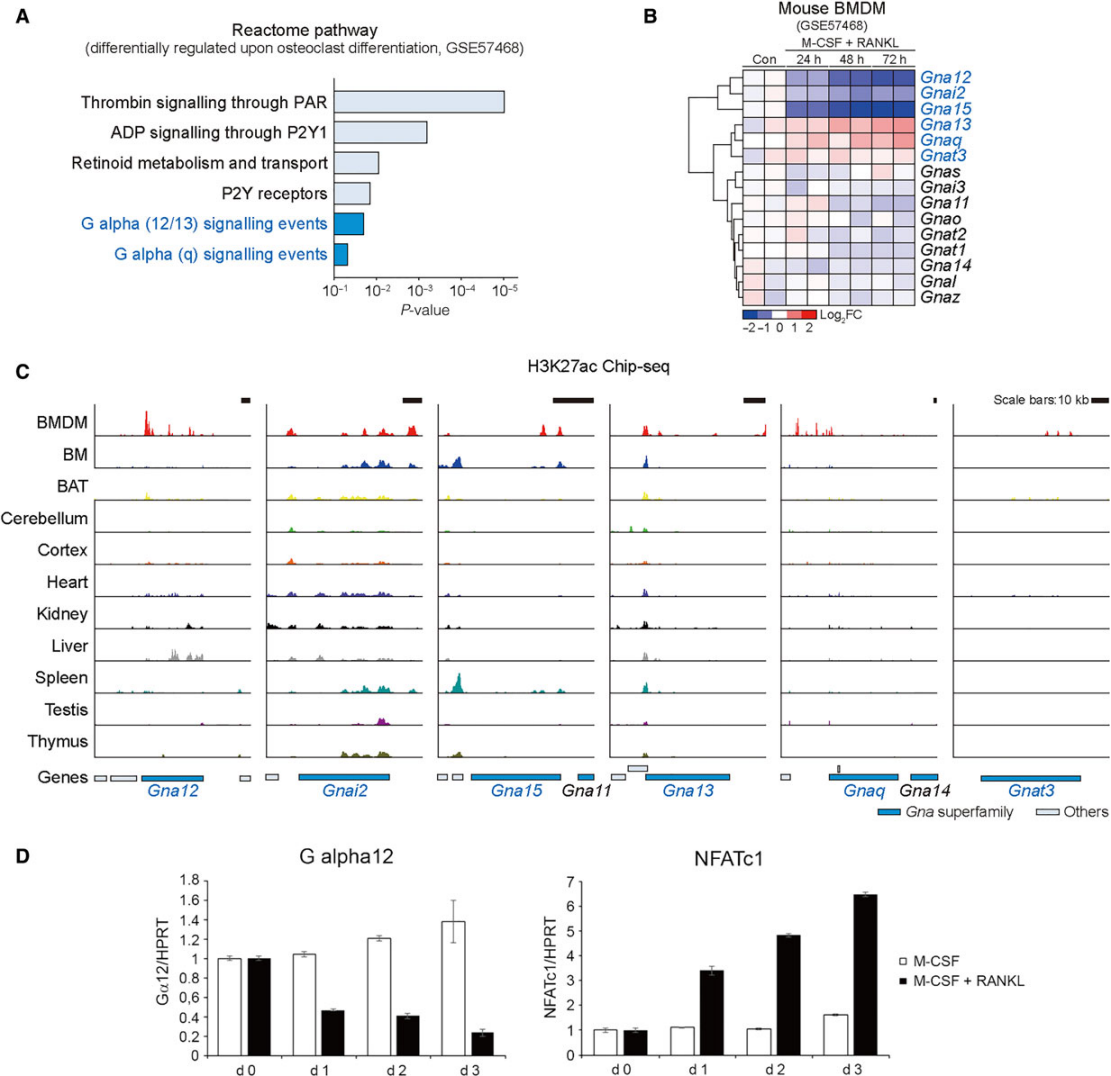
Representative Gα transcript levels during osteoclast differentiation. (**A**) Enriched pathway analysis using the database of the Reactome pathways (GSE57468). (**B**) Heatmap and hierarchical correlation analyses for the expression of Gα genes (GSE57468). Red, up‐regulation; blue, down‐regulation. (**C**) H3K27ac ChIP‐seq analyses on the Gα genes using different tissues. The y axis corresponds to ChIP‐seq signal intensity in a range of 0 to 52 (Read Per Million, RPM). Gα gene bodies were indicated as blue. (**D**) Bone marrow‐derived macrophages (BMMs) were cultured with or without RANKL (120 ng/ml) in the presence of M‐CSF (30 ng/ml) for 0, 1, 2 or 3 days. The mRNA level of Gα12 and NFATc1, a marker of osteoclast differentiation, was analysed by RT‐qPCR. BMDM, bone marrow‐derived macrophages; BM, bone marrow; BAT, brown adipose tissue.

To explore the putative signalling pathway responsible for the regulation of bone homeostasis, we first analysed a microarray data set obtained using the mouse bone marrow‐derived macrophages (BMDMs), primary cells of osteoclast precursor. We found that expression of genes involved in several G protein‐coupled receptor pathways changed during osteoclast differentiation triggered by M‐CSF and RANKL treatment (Fig. 1A). Along with Gα12/13 and Gαq signalling events, PAR and P2Y1 signalling pathways shown to link to Gα12/13 or Gαq showed significant changes. Next, we analysed Gα transcript levels; Gα12 transcript levels were significantly down‐regulated along with those of Gαi2 and Gα15, whereas Gα13, Gαq or Gαt3 transcript levels were rather increased (Fig. 1B). We then analysed the profile of histone modification on each gene and the surrounding regions in BMDMs and other tissues using H3K27ac signals and found that *Gna12, Gnai2, Gna15, Gna13 and Gnaq* DNA regions were transcriptionally active in BMMs, whereas *Gnat3* DNA regions were not (Fig. 1C, and Fig. S1). The H3K27ac signals on the *Gna12* DNA regions were strongest in BMMs among the tissues examined, suggestive of the tissue‐specific role of Gα12 in osteoclast differentiation. Consistently, Gα12 mRNA levels were decreased by M‐CSF and RANKL treatment in our real‐time PCR analyses (Fig. 1D). In contrast, Gα12 mRNA levels were not changed by M‐CSF treatment alone. Increases in NFATc1 mRNA levels confirmed successful osteoclast differentiation (Fig. 1D). These results led us to further explore the role of Gα12 in osteoclast differentiation and bone homeostasis.

### Gα12 knockout mice show osteopetrotic phenotype

**Figure 2:**
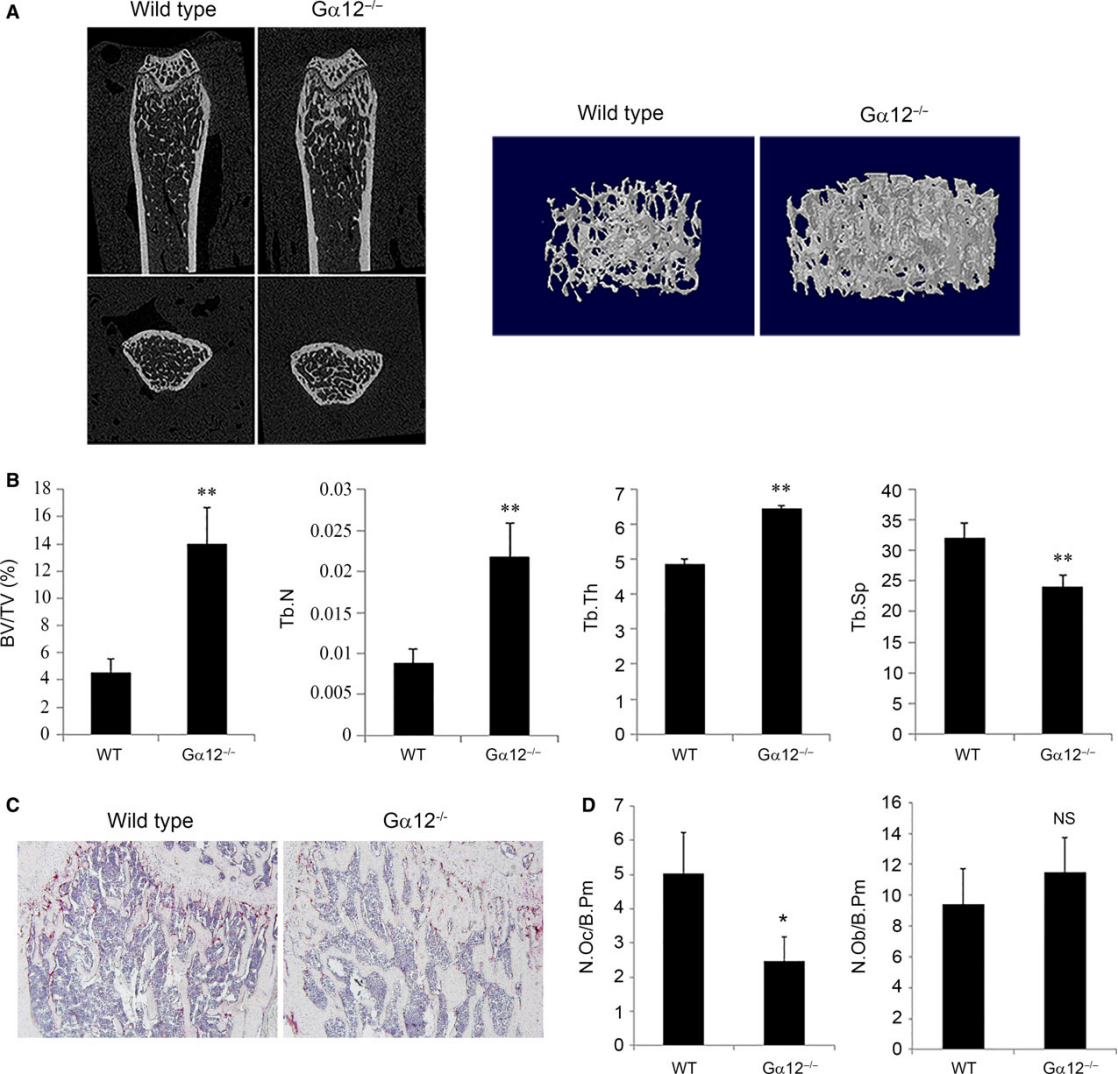
Gα12‐knockout mice show osteopetrotic phenotype. (**A** and **B**) Femurs from 9‐week‐old WT and Gα12^−/−^ male mice were subjected to μCT analysis. (**A**) Representative coronal and horizontal images and three‐dimensional images of trabeculae of WT and Gα12^−/−^ mice are shown. (**B**) Bone parameters of trabecular bone volume per tissue volume (BV/TV), trabecular thickness (Tb.Th), trabecular number (Tb.N) and trabecular separation (Tb.Sp) were analysed with a μCT analysis program (*n* = 4 per group). (**C**) For histological analysis, decalcified femurs were sectioned and stained for TRAP activity and counter‐stained with haematoxylin. (**D**) Osteoclasts and osteoblasts were counted with Osteomeasure software. NS, non‐significance *versus* WT. N.OC, number of osteoclasts; N.OB, number of osteoblasts; B.Pm, bone perimeter. Data are presented as means ± standard deviations (S.D.). *P < 0.05; **P < 0.005.

To evaluate the potential role of Gα12 in bone metabolism, femurs from 9‐week‐old Gα12‐knockout (Gα12^−/−^) mice were analysed by micro‐computed tomography (μCT). The trabecular bone volume (BV) of the Gα12^−/−^ mice was about threefold higher than that of the wild‐type (WT) mice (Fig. 2A and B). The trabecular number (Tb.N) and trabecular thickness (Tb.Th) were also higher, while trabecular separation (Tb.Sp) was lower in the Gα12^−/−^ mice compared to the WT mice (Fig. 2B). To investigate whether the increment in bone mass was caused by changes in the population of bone cells, we assessed the numbers of osteoclasts and osteoblasts by histological methods. With increased trabecular bone area, we observed decreased numbers of osteoclasts in the Gα12^−/−^ bone tissue sections (Fig. 2C and D). However, the numbers of osteoblasts were not significantly different between the WT and Gα12^−/−^ mice (Fig. 2D). The μCT analyses of 15‐week‐old mice also revealed a similar osteopetrotic phenotype in Gα12^−/−^ femurs (Fig. S2). These observations suggest that Gα12 plays a role in bone metabolism, perhaps by regulating osteoclastogenesis.

### Gα12 depletion impairs osteoclast differentiation and bone resorptive function

**Figure 3:**
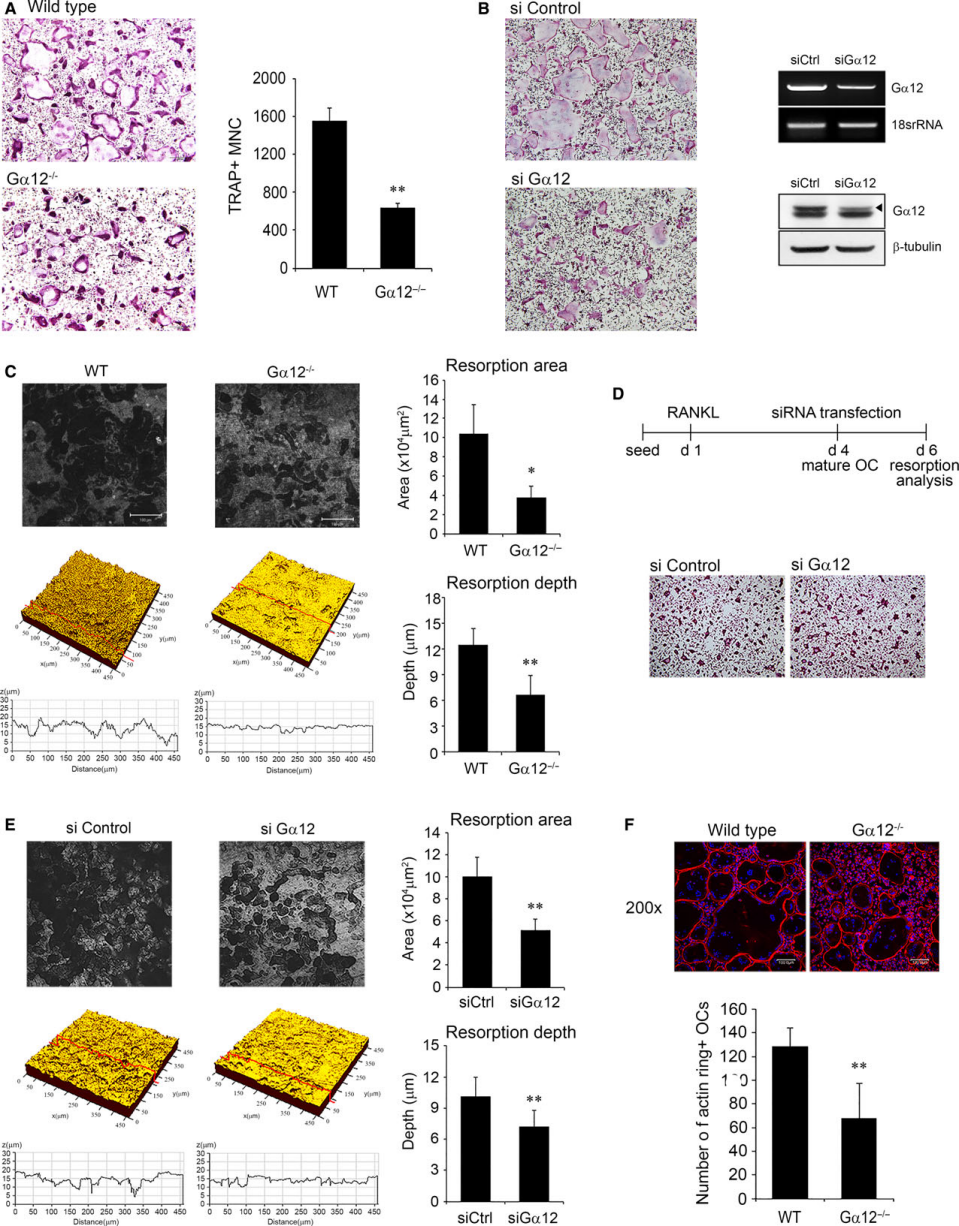
Gα12 depletion impairs osteoclast differentiation and bone resorptive function. (**A**) Bone marrow‐derived macrophages (BMMs) of WT or Gα12^−/−^ mice were cultured with 30 ng/ml M‐CSF and 120 ng/ml RANKL for three days and stained for TRAP. Images were captured using a light microscope (magnification 100 ×), and TRAP‐positive multinucleated cells (MNCs) were counted. (**B**) BMMs were transfected with 40 nM of negative control or Gα12‐specific siRNA and cultured with M‐CSF and RANKL for three days. Images of TRAP‐stained cells are shown, and knockdown was confirmed by RT‐PCR (right upper) and Western blotting (right lower). (**C**) BMMs were cultured on dentin slices for nine days with RANKL and M‐CSF. The dentin slices were observed using a confocal microscope. Bone resorption area and depth were measured with image analysis software. (**D**) BMMs were seeded on dentin slices and cultured with M‐CSF and RANKL for 4 days. After siRNA transfection at day 4, the cells were further cultured for additional 2 days. After removing the cells, dentin surface was analysed using a confocal microscope. (**E**) Bone resorption area and depth were measured with image analysis software. (**F**) BMMs were cultured with M‐CSF and RANKL for 4 days, and actin was labelled with rhodamine‐phalloidin (red). Nuclei were counter‐stained with DAPI. Images were captured using a confocal microscope (magnification 200 ×), and actin ring‐positive osteoclasts were counted. Data are presented as means ± S.D. *P < 0.05; **P < 0.005.

After the μCT and histologic analyses, we assessed the function of Gα12 in osteoclast differentiation. BMMs were prepared from WT and Gα12^−/−^ mice, and the cells were cultured with osteoclastogenic medium containing M‐CSF and RANKL. On day 3 after culturing, WT cells formed a considerable number of multinucleated cells that were positive for the osteoclast marker TRAP. However, <50% of multinucleated osteoclasts were generated in the Gα12^−/−^ group (Fig. 3A). A similar pattern was observed with the BMMs, in which Gα12 expression was reduced with a siRNA‐mediated knockdown system (Fig. 3B).

Next, we examined the bone resorptive function of Gα12^−/−^ osteoclasts. Osteoclasts were cultured on dentin slices, and the area and depth of the resorption pits were measured. Compared to the WT osteoclasts, the Gα12^−/−^ osteoclasts generated smaller and shallower pits (Fig. 3C). To specify the reduced resorption is due to impaired resorption activity or decreased osteoclast differentiation, we tested resorption activity of mature osteoclasts. Gα12‐specific siRNAs were introduced to osteoclasts generated by culturing BMMs for 4 days with RANKL (Fig. 3D). Gα12 knockdowned osteoclasts showed significantly diminished resorption area and resorption depth (Fig. 3E) when the degree of osteoclast differentiation was similar to that of the control cells (Fig. 3D). It implies that Gα12 is involved not only in the osteoclastic differentiation but also in the resorption activity of mature osteoclasts.

We also assessed actin ring formation, because the actin ring is a functionally important cytoskeletal structure for bone resorption and a typical morphological feature of active mature osteoclasts. As expected, we observed a reduced number of osteoclasts containing actin rings in Gα12^−/−^ group compared to the WT group (Fig. 3F). However, the actin ring morphology in mature osteoclasts did not show evident differences between the WT and Gα12^−/−^ mice.

Taken together, these results indicate that Gα12 depletion impaired RANKL‐induced osteoclast differentiation and bone resorption.

### Gα12 regulates osteoclastogenesis *via* NFATc1

**Figure 4:**
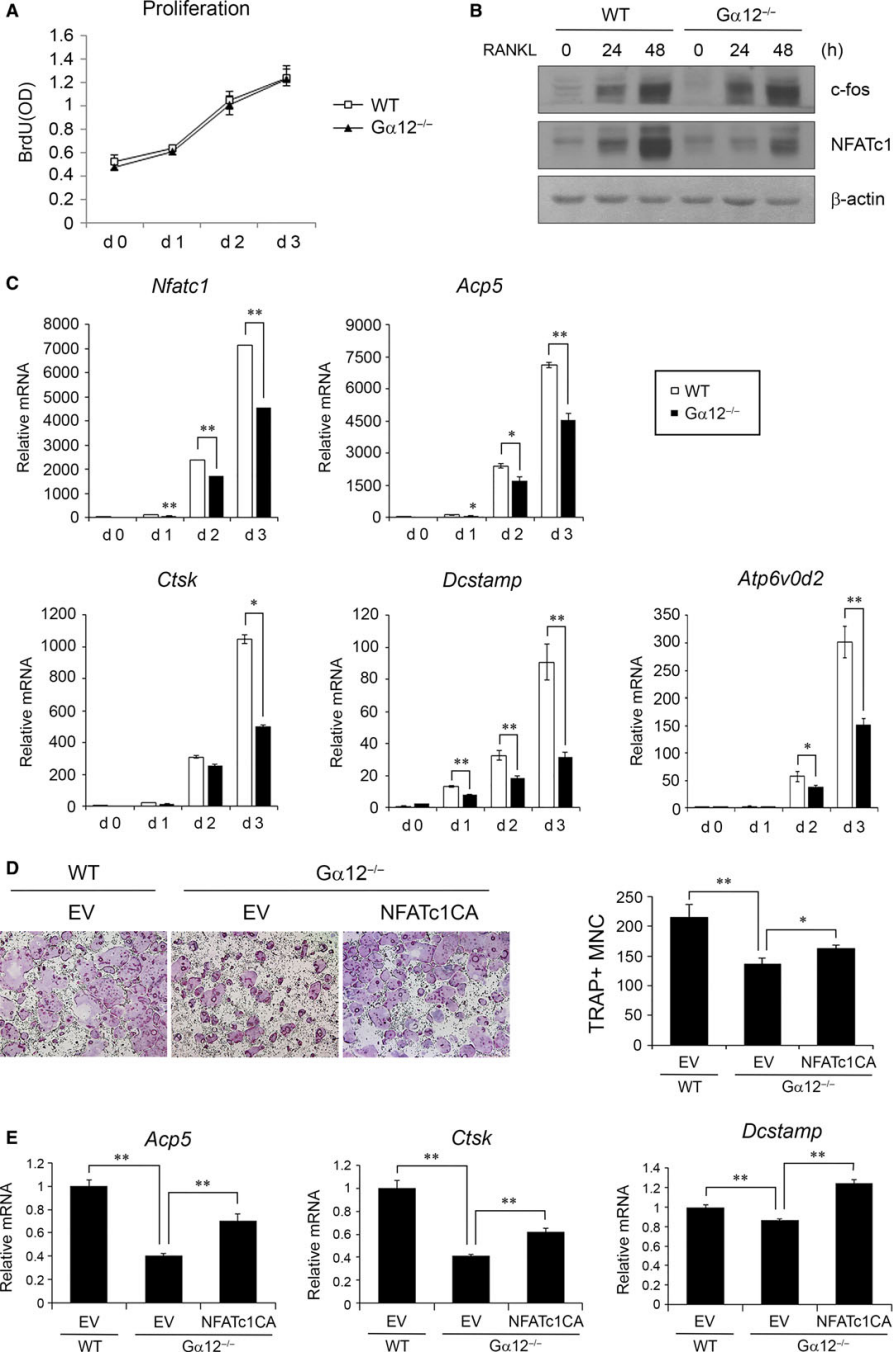
Gα12 regulates osteoclastogenesis *via* NFATc1. (**A**) WT or Gα12^−/−^ bone marrow‐derived macrophages (BMMs) were cultured in 96‐well plates with RANKL and M‐CSF, and BrdU assays were performed on days 0, 1, 2 and 3. (**B**) Protein lysates were prepared at 0, 24 and 48 hrs after stimulation by RANKL in the presence of M‐CSF. Western blot analysis was performed using antibodies for c‐Fos, NFATc1 and β‐actin. (**C**) BMMs from WT mice or Gα12^−/−^ mice were cultured with M‐CSF and RANKL for three days. The expression levels of NFATc1 and NFATc1‐driven genes were analysed by real‐time PCR. (**D**) NFATc1CA was overexpressed in BMMs by retroviral transduction and cultured with M‐CSF and RANKL for 6 days (magnification 40 ×). TRAP+ MNCs were counted after TRAP staining. (**E**) After retroviral transduction of NFATc1CA, the expression levels of osteoclast marker genes were analysed by real‐time PCR at day 2 after RANKL treatment. EV, empty vector; CA, constitutively active. Data are presented as means ± S.D.. *P < 0.05; **P < 0.005.

Gα12 has been implicated in oncogenic transformation and proliferation in other cell types, mainly fibroblast cell line 8, 21. To examine whether Gα12 promotes osteoclast differentiation by increasing the proliferation of osteoclast precursors, we performed a bromodeoxyuridine (BrdU) cell proliferation assay. However, there was no difference in proliferation between WT and Gα12^−/−^ BMMs during osteoclast differentiation (Fig. 4A).

NFATc1 is a key transcription factor in osteoclastogenesis and mediates the expression of many essential genes for osteoclast differentiation 4. To investigate whether Gα12 regulates NFATc1 expression during RANKL‐induced osteoclastogenesis, we measured the expression level of NFATc1 in Gα12^−/−^ BMMs. NFATc1 protein level was lower in Gα12^−/−^ BMMs compared to that in WT BMMs at 24 and 48 hrs after RANKL stimulation (Fig. 4B). Sequentially, the induction of osteoclast‐specific genes that are direct targets of NFATc1, such as Acp5, Ctsk, Dcstamp and Atp6v0d2, was also much less prominent in the Gα12^−/−^ BMMs than in the WT BMMs during RANKL‐induced osteoclast differentiation (Fig. 4C). However, c‐Fos, which is one of the transcription factors inducing NFATc1, did not differ between the two groups at protein level (Fig. 4B). To clarify that Gα12 regulates osteoclast differentiation through NFATc1, we overexpressed constitutive active (CA) NFATc1 in Gα12^−/−^ BMMs using a retroviral system. NFATc1CA overexpression partially recovered the generation of osteoclasts from Gα12^−/−^ BMMs (Fig. 4D) and the expression of osteoclast marker genes (Fig. 4E). These results indicate that Gα12 regulates osteoclastogenesis by modulating NFATc1 expression and, consequently, the expression of NFATc1‐driven genes.

### Gα12 affects BMM migration and osteoclast resorption through Rho activation

**Figure 5:**
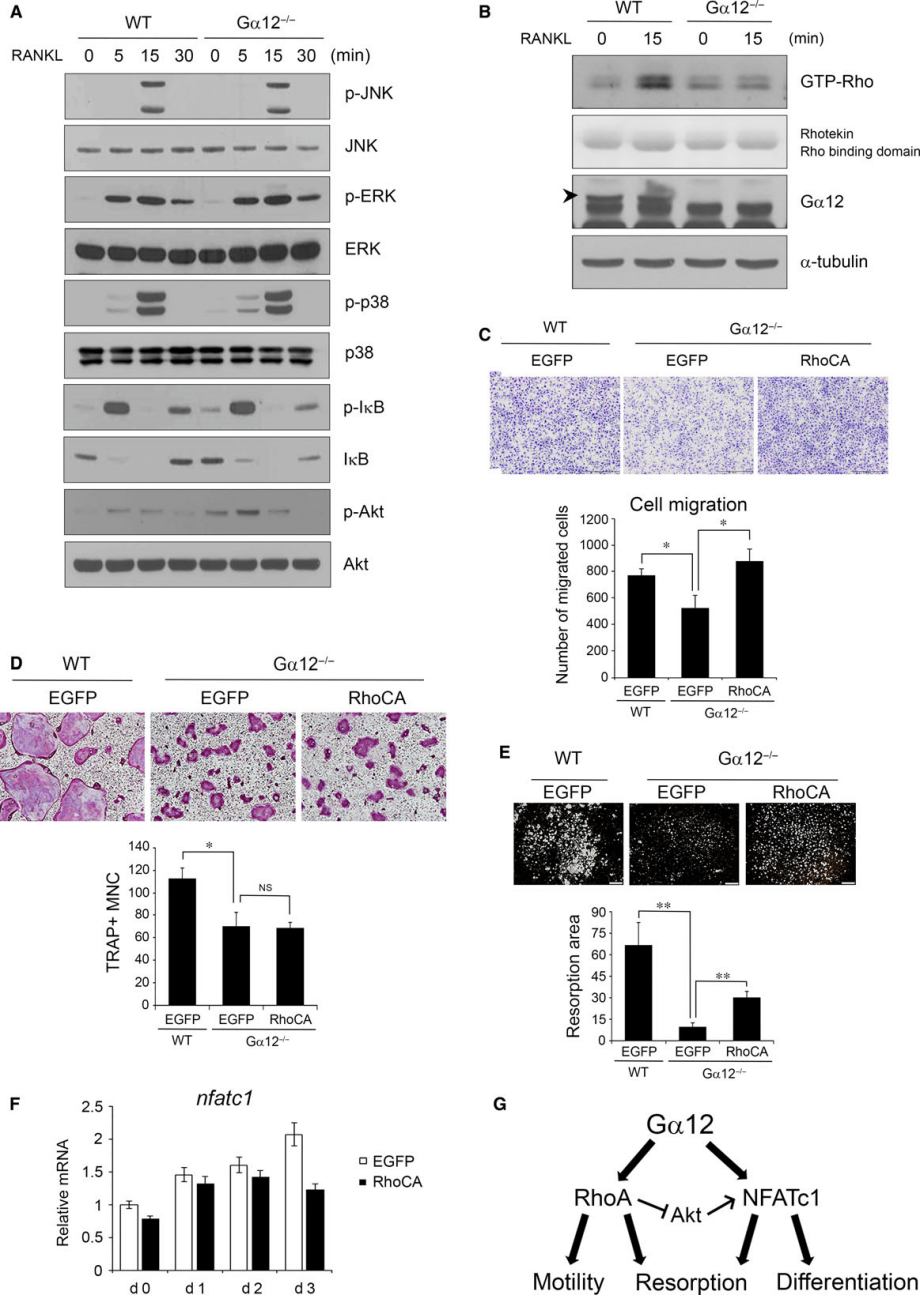
Gα12 regulates precursor migration and bone resorption through mediating Rho activation by RANKL. (**A**) Bone marrow‐derived macrophages (BMMs) were serum‐starved for 4 hrs and then stimulated with 600 ng/ml RANKL. Stimulated cells were harvested after 0, 5, 15 and 30 min., and protein lysates were prepared. Western blot analysis was performed using antibodies against the phospho forms or total forms of the indicated proteins. (**B**) BMMs were serum‐starved for 3 hrs, and proteins were harvested at 0 and 15 min. after RANKL stimulation. After GTP‐Rho was precipitated, immunoblotting was performed using anti‐Rho antibody. For a loading control, the Rhotekin Rho‐binding domain used for pull‐down was stained with Ponceau S after transfer to membranes. (**C**) Transwell migration assays were performed for 16 hrs using pcDNA3‐EGFP‐ or RhoCA‐transfected WT or Gα12^−/−^ BMMs. Migrated cells were stained with crystal violet and counted. (**D**) BMMs transfected with pcDNA3‐EGFP‐ or RhoCA were cultured with M‐CSF and RANKL for 6 days and TRAP+ MNC were counted (magnification 40x). (**E**) Transfected BMMs were cultured with M‐CSF and RANKL on calcium phosphate‐coated plates for 6 days. Remained calcium phosphate was stained by vonKossa, and resorption area is quantified with ImageJ software. (**F**) Transfected BMMs were cultured with M‐CSF and RANKL for 3 days, and NFATc1 mRNA levels were analysed by real‐time PCR. (**G**) Schematic diagram on the role of Gα12 for osteoclast differentiation and bone resorption. NS, not significant. Data are presented as means ± S.D.. *P < 0.05; **P < 0.005.

To define the molecular mechanism involved in osteoclast regulation by Gα12, we tested signalling pathways that have been reported to be activated by RANKL. BMMs were stimulated with RANKL, and the phosphorylation of MAPK, IκB and Akt was assessed by Western blotting. In the Gα12^−/−^ and WT cells, the RANKL‐mediated activation of ERK, JNK, and p38 MAPK was detected at similar levels, and the activity of canonical NF‐κB signalling reflected as IκB phosphorylation, and degradation was also not significantly different between Gα12^−/−^ and WT BMMs (Fig. 5A). However, Akt phosphorylation was increased in Gα12^−/−^ BMMs (Fig. 5A).

Gα12 has been well described to have RhoGEF as its effector molecule and to regulate cellular migration and polarity through Rho signalling activation 22, 23. To test whether Gα12 affects Rho activation in osteoclasts, we performed Rho‐GTP pull‐down assays. By RANKL stimulation, the level of GTP‐bound RhoA (the active form of RhoA) was substantially increased after 15 min. in WT BMMs. In contrast, the Gα12^−/−^ BMMs showed only a slight increase in GTP‐RhoA (Fig. 5B). To demonstrate that Gα12 affects the migration of osteoclast precursor cells through RhoA activation, we evaluated the migrating ability of Gα12^−/−^ BMMs *versus* WT BMMs. Gα12^−/−^ BMMs showed a decreased migration level (32.4 ± 8.4%), and this decline was rescued by the overexpression of constitutively active RhoA (RhoCA) (Fig. 5C).

We next tested whether the overexpression of RhoCA could recover osteoclast formation from and NFATc1 expression in Gα12^−/−^ BMMs. As shown in Figure 5D, osteoclastogenesis was not influenced by RhoCA overexpression. Furthermore, NFATc1 expression was not elevated, rather inhibited at a later stage, by RhoCA overexpression (Fig. 5F). However, the impaired resorption capacity of Gα12^−/−^ osteoclasts was partially restored by RhoCA overexpression (Fig. 5E). Taken together, these results indicate that Gα12 regulates BMM migration and bone resorption through RhoA activation while influencing osteoclast differentiation through NFATc1 up‐regulation.

## Discussion

By μCT analyses, we found increased trabecular bone volume in Gα12^−/−^ mice compared to that in WT mice. This observation suggests that Gα12 has a significant role in bone homeostasis. Histological experiments with femoral tissues revealed that Gα12 deficiency selectively affected osteoclast numbers with little effect on osteoblasts. In addition to, *in vitro* cultures also showed Gα12 deficiency impairs osteoclast differentiation. These results seem to result from the decreased expression of NFATc1, the master transcription factor of osteoclastogenesis.

A well‐established downstream effector of Gα12 is monomeric GTPase RhoA 22. RhoA regulates cytoskeletal dynamics, cell movement and divergent cellular functions. In osteoclasts, it has been reported that RhoA is crucial for osteoclast podosome organization, motility and bone resorption 24, 25. In our experiments, Gα12^−/−^ BMMs showed decreased RhoA activity, which caused a reduction in osteoclast precursor migration and bone resorption (Fig. 5C and E). However, overexpression of the constitutively active RhoA could not recue impaired osteoclast formation by Gα12 deficiency (Fig. 5D). It implies that RhoA is more crucial for the precursor migration and resorption activity than for the differentiation in Gα12‐mediated osteoclast control. Nonetheless, indirect contribution of RhoA to acceleration of osteoclast formation by stimulating recruitment of precursors to bone surface *in vivo* is still conceivable.

In investigations on the RANKL‐induced signalling, we found that Gα12 deficiency increased Akt phosphorylation (Fig. 5A). RhoA has been reported to inhibit Akt 26, and the Akt‐dependent GSK3β‐NFATc1 pathway has been suggested to positively regulate osteoclastogenesis 27. Therefore, one may expect that RhoA activation by Gα12 leads to attenuation of NFATc1 expression. Indeed, we detected slightly down‐regulated NFATc1 expression by RhoCA overexpression (Fig. 5F). However, Gα12^−/−^ cells consistently displayed elevated osteoclastogenesis and NFATc1 expression (Figs 2, 3, 4). Based on our findings and results shown in previous reports, we propose that RhoA activation by Gα12 increases cell mobility and bone resorption activity while negatively acting on NFATc1 *via* Akt inhibition. However, the direct positive effect of Gα12 on NFATc1 overrides the NFAT attenuation by RhoA, leading to enhanced osteoclastogenesis (Fig. 5G). Further studies are required to reveal the molecular mechanism for NFATc1 up‐regulation by Gα12 in osteoclasts.

Through Gα transcript analysis, we found *Gna12* expression is down‐regulated during osteoclastogenesis, and it was verified by our real‐time PCR analyses. Although the molecular mechanism of Gα12 suppression during osteoclastogenesis was not investigated in this study, we could find, using a program for prediction of transcription factor binding sites, several transcription factors that were supposed to bind the Gα12 promoter region. Among them, Pax6, a transcriptional repressor of which expression is increased during osteoclastogenesis 28 and Foxo1 and ATF3, transcriptional activator of which expression is decreased during osteoclastogenesis 29, 30 may be attributed to the down‐regulation of Gα12 during RANKL‐induced osteoclastogenesis.

Although it is apparent that Gα12 modulates RANKL‐induced osteoclastogenesis, whether Gα12 is activated directly through RANKL stimulation is not clear. Considering that Gα12 is activated by its binding to GPCR, Gα12 might be activated through some GPCR ligands secreted from osteoclasts during RANKL‐induced osteoclastogenesis. More than 30 GPCRs, including P2Y receptor and sphingosine‐1‐phaophate receptor, have been reported to couple to either Gα12, Gα13 or both, and these GPCRs have various ligands or agonists 31. In RANKL‐induced osteoclastogenesis, the identification of the GPCR that acts as the main upstream mediator of Gα12 requires further study. Furthermore, the Gα12‐mediated signalling pathway functionally interacts with not only Gα13, but also Gα_q/11_‐mediated signalling systems 18, 32. This complexity makes it difficult to distinguish contribution of each pathway from that of the others to the regulation of osteoclastogenesis.

In our histological analysis, no difference in osteoblast numbers between Gα12^−/−^ and WT femurs was observed. There have been some reports that suggest Gα12 activation by parathyroid hormone (PTH) in osteoblastic cells can regulate osteoclastogenesis. In UMR‐106 osteoblastic cells, Gα12 was shown to mediate PTH‐stimulated phospholipase D activation through Rho 33, and in turn, phospholipase D activates osteoclastogenesis through IL‐6 production 34. In the coculture of UMR‐106 and RAW264.7 cells, active Gα12 reduced the ratio of RANKL/osteoprotegerin production from UMR‐106 cells in response to PTH and inhibited the TRAP activity of RAW264.7 cells 35. These *in vitro* studies proposed both positive and negative roles of Gα12 in osteoblasts to affect osteoclastogenesis. However, the *in vivo* implication of these studies is not clear. Considering that the crosstalk between osteoblasts and osteoclasts is a very important feature in bone physiology, studies with Gα12^−/−^ mice under various conditions, including ovariectomized or hormone‐stimulated conditions, will be helpful for exploring the function of Gα12 in bone metabolism. Meanwhile, the roles of Gα12 in various human diseases have been recognized 36. Our results suggest the possibility of involvement of Gα12 in the pathophysiology of bone diseases like osteoporosis or rheumatoid arthritis.

Here, for the first time, we demonstrated that the loss of Gα12 expression results in abnormally augmented trabecular bone volume, coincident with a decrease in osteoclast numbers. We also revealed that Gα12 regulates osteoclastogenesis *via* NFATc1 along with modulating osteoclast migration and resorption through RhoA activation.

## Conflicts of Interest

The authors declare no conflict of interests.

## Supporting information


**Figure S1** Gα transcript levels during osteoclast differentiation.

**Figure S2** Bone analysis in 15‐week‐old Gα12^−/−^ male mice.
