# Supplementary figures and images for: Gα12 regulates osteoclastogenesis by modulating NFATc1 expression
Source: J Cell Mol Med. 2017 Oct 27;22(2):849–60. doi: 10.1111/jcmm.13370 (PMC5783869; doi:10.1111/jcmm.13370)

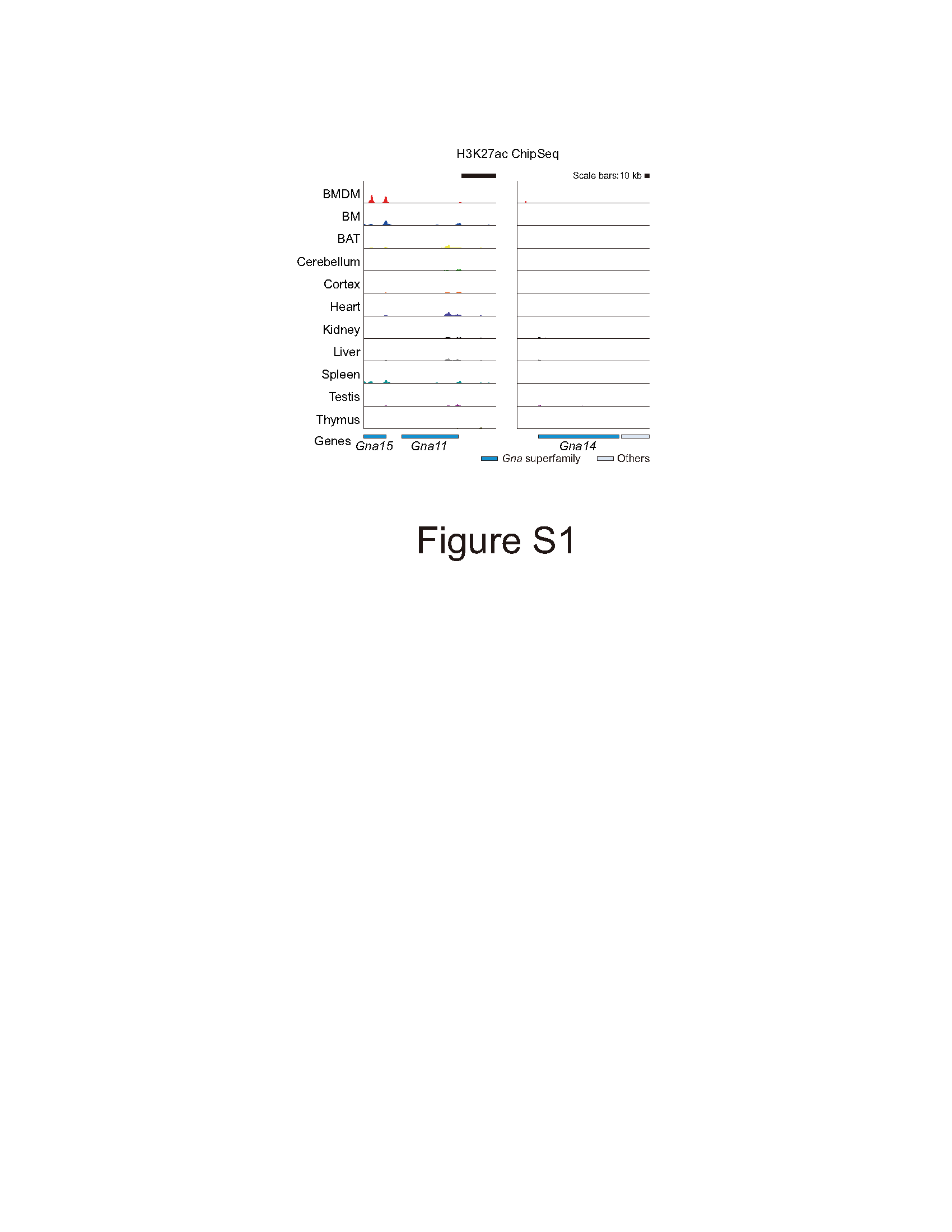

Supplement: Supplementary file 1 — Figure S1 Gα transcript levels during osteoclast differentiation. [file JCMM-22-849-s001.tif]

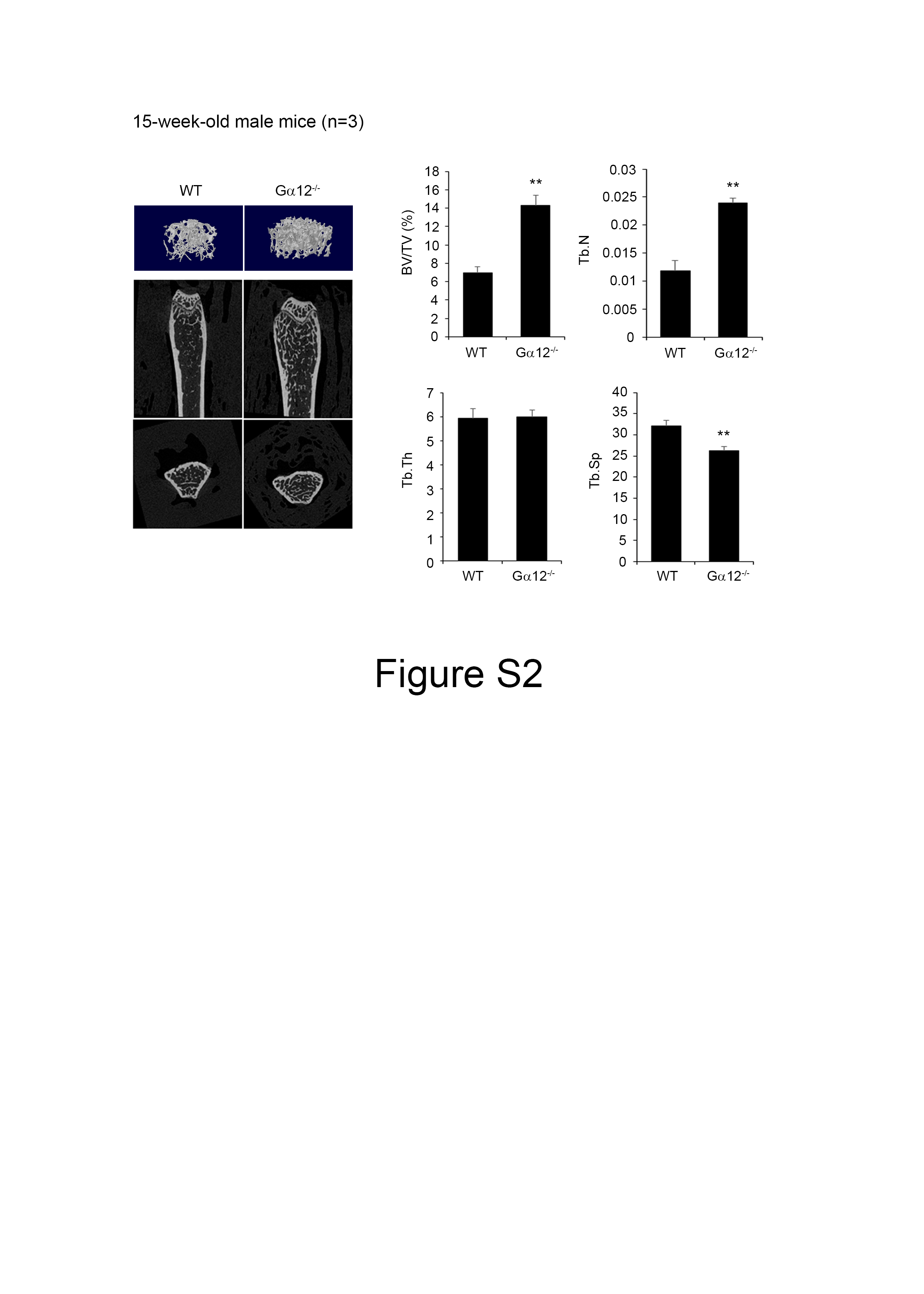

Supplement: Supplementary file 2 — Figure S2 Bone analysis in 15‐week‐old Gα12−/− male mice. [file JCMM-22-849-s002.tif]
