# Supplementary material for: Gα12 regulates osteoclastogenesis by modulating NFATc1 expression
Source: J Cell Mol Med. 2017 Oct 27;22(2):849–60. doi: 10.1111/jcmm.13370 (PMC5783869; doi:10.1111/jcmm.13370)
Supplement: Supplementary file 3 [file JCMM-22-849-s003.docx]

**Figure S1. G**α **transcript levels during osteoclast differentiation**

H3K27ac ChIP-seq analyses at the *Gna11* and *Gna14* loci. The y axis corresponds to ChIP-seq signal intensity in a range of 0 to 52 (Read Per Million, RPM). Gα gene bodies were shown as blue.

**Figure S2. Bone analysis in 15-week-old Gα12^-/-^ male mice**

Femurs from 15-week-old WT and Gα12^-/-^ male mice were subjected to μCT analysis. Left: representative coronal and horizontal images and three-dimensional images of trabeculae of WT and Gα12^-/-^ mice are shown. Right: bone parameters of trabecular bone volume per tissue volume (BV/TV), trabecular thickness (Tb.Th), trabecular number (Tb.N), and trabecular separation (Tb.Sp) were analyzed with a μCT analysis program (n = 3 per group). Data are presented as means ± SDs. **p < 0.005
